# Polyploidization Increases the Lipid Content and Improves the Nutritional Quality of Rice

**DOI:** 10.3390/plants11010132

**Published:** 2022-01-04

**Authors:** Wei Wang, Qiang Tu, Rongrong Chen, Pincang Lv, Yanqing Xu, Qian Xie, Zhaojian Song, Yuchi He, Detian Cai, Xianhua Zhang

**Affiliations:** School of Life Sciences, Hubei University, Wuhan 430062, China; 201911110711068@stu.hubu.edu.cn (W.W.); 201811110711049@stu.hubu.edu.cn (Q.T.); 201911110711104@stu.hubu.edu.cn (R.C.); 202111107010084@stu.hubu.edu.cn (P.L.); 202021107011124@stu.hubu.edu.cn (Y.X.); 202021107011115@stu.hubu.edu.cn (Q.X.); 20050040@hubu.edu.cn (Z.S.); 20040671@hubu.edu.cn (Y.H.); 20040640@hubu.edu.cn (D.C.)

**Keywords:** LC-MS/MS, nutritional components, tetraploid rice, widely targeted metabolomics

## Abstract

Plant polyploidization is frequently associated with changes in nutrient contents. However, the possible contribution of metabolites to this change has not been investigated by characterizing the metabolite contents of diploid and tetraploid forms of rice (*Oryza sativa* L.). We compared the metabolites of a group of diploid–tetraploid *japonica* brown rice and a group of diploid–tetraploid *indica* brown rice based on liquid chromatography–tandem mass spectrometry. In total, 401 metabolites were identified; of these, between the two diploid–tetraploid groups, 180 showed opposite expression trends, but 221 showed the same trends (147 higher abundance vs. 74 lower abundance). Hierarchical cluster analysis of differential metabolites between diploid and tetraploid species showed a clear grouping pattern, in which the expression abundance of lipids, amino acids and derivatives, and phenolic acids increased in tetraploids. Further analysis revealed that the lipids in tetraploid rice increased significantly, especially unsaturated fatty acids and phospholipids. This study provides further basis for understanding the changes in rice nutritional quality following polyploidization and may serve as a new theoretical reference for breeding eutrophic or functional rice varieties via polyploidization.

## 1. Introduction

Rice (*Oryza sativa* L.) is one of the most important food crops in the world. While exploring increases in rice yield, the improvement of rice nutritional quality is also pursued [1]. Rice grains are mainly composed of starch (85–90%), protein (7–12%), lipid (0.3–3%), mineral elements, and vitamins [2,3]. Although the lipid content of rice is low, it has high nutritional value and health care function [4]. The lipids of rice are divided into non-starch lipids (NSLs) and starch lipids [5]. The NSLs refer to the lipids on the surface of starch granules, which mainly exist in the form of spherical fat bodies [6]. The NSLs mainly include glycerides, triglycerides, phospholipids (PLs), glycolipids, and free fatty acids [7], as well as oryzanol, tocopherol, phenolic acid, phytosterol, squalene, and other physiologically active substances [8,9]. The main free fatty acids in rice are oleic (C18:1), linoleic (C18:2), palmitic (C16:0), stearic (C18:0), myristic (C14:0), palmitoleic (C16:1), linolenic (C18:3), and arachidonic (C20:4) acids [10,11,12,13]. Most of the free fatty acids in rice are high-quality unsaturated fatty acids, among which arachidonic, linolenic, and linoleic acids have obvious effects in preventing arteriosclerosis and hypercholesterolemia [14]. Low odor threshold volatile compounds, which can be derived from the oxidation of unsaturated fatty acids, are more abundant in the fragrant rice varieties. Thus, the unsaturated fatty acids contribute to rice fragrance, and thereby to overall quality [15]. Compared with starch and protein, increasing the content of unsaturated fatty acids can significantly improve rice-eating quality [16]. In addition, PLs, another important component of rice NSLs, have important nutritional value. They are important polar lipids, and exist widely in bacteria, animals, and plants [4]. Due to their important biochemical functions, many studies have found that dietary PLs are an important way to prevent diabetes, coronary heart disease, inflammation, cancer, and other chronic diseases [17]. Therefore, increasing the lipid content in rice is a way to breed good-quality and nutrient-rich varieties.

Polyploids are widely distributed in nature [18,19,20,21]. Almost all eukaryotes and most angiosperms, as well as crops such as rice, have experienced polyploidization in the evolutionary process [22,23,24]. Polyploid rice has some advantageous agronomic traits, such as large grain sizes, high 1000-grain weights, strong stem, and strong stress tolerances [25]. However, their production and use have been restricted by many factors, especially low seed-setting rates [26,27]. In recent years, highly fertile tetraploid rice lines with polyploid meiotic stability (*PMeS*) genes and neo-tetraploid rice lines with higher than 80% seed-setting rates have been bred to address this restriction [28,29,30]. Subsequently, a series of theoretical and applied studies on high fertility tetraploid rice was carried out [31,32,33,34,35], which promotes the application of polyploid rice. Polyploidization not only causes gigantism, leading to enhanced biomass production, it also changes nutritional quality; for example, contents of carbohydrates, proteins, vitamins, and alkaloids generally increase [36,37]. After polyploidization, the nutritional quality of rice also changes [38]. Compared with diploid rice, the protein content of autotetraploid rice increases by about 30%, and amino acid content increases by 20–30%, but the amylose content decreases by about 12% [25]. A recent investigation revealed that polyploidization increases the glutelin content of rice seeds by influencing glutelin biosynthesis, transport, and deposition [39]. These changes improved the nutrition and palatability of rice [38]. In the past, the changes in nutrients after polyploidization have usually been analyzed using traditional chemical methods. However, in light of the central role played by metabolism in nutrition, metabolomics is rapidly being established as a key analytical method in nutritional studies [40]. Metabolomics is a method of qualitatively and quantitatively analyzing all the metabolites in an organism, and it can be used to explore dynamic changes in metabolites, as well as the accumulation patterns and genetic origins of plant metabolites [41,42]. Widely targeted metabolomics analysis is a novel approach that can simultaneously quantify hundreds of known metabolites and nearly 1000 known and unknown metabolites [43,44,45,46]. Now, metabonomics is being increasingly used to analyze rice nutrients. Hu et al. compared 121 metabolites in mature seeds of a wide panel of *japonica* and *indica* cultivars, laying a foundation for improving rice quality through metabolic engineering [47]. Later, they investigated the dynamic metabolic changes during the rice grain development of two *japonica* and two *indica* cultivars using a non-targeted metabolomics approach [48]. In addition, analyses of rice by gas chromatography-MS have revealed several odor-active volatile compounds that may provide the characteristic aromas of different rice varieties [15,49,50]. The secondary metabolites of the Chinese and North American wild rice have been analyzed using an UHPLC-QqQ-MS-based metabolomics approach, providing reference values for the isolation and identification of functional compounds from wild rice [51]. However, to the best of our knowledge, there has been no metabonomics report of the effect of polyploidization on rice nutrition. Here, we compared the metabolites of diploid and tetraploid rice based on liquid chromatography-tandem mass spectrometry (LC-MS/MS). The metabolite expressions among two rice lines with two ploidy levels were analyzed and the significantly different metabolites were identified. Our study provides a basis for using polyploidization to change rice nutritional quality and serves as a new theoretical reference for breeding nutrient-rich rice varieties via polyploidization.

## 2. Materials and Methods

### 2.1. Rice Samples

Diploid rice ‘Balilla’ (*O. sativa* ssp. *japonica*, 2n = 2*x* = 24) is a *japonica* rice variety from Italy. Diploid rice ‘Yangdao 6’ (*O. sativa* ssp. *indica*, 2n = 2*x* = 24) is an *indica* rice variety, and was bred by Hongxi Zhang (Institute of Agricultural Sciences for Lixiahe Region in Jiangsu, Yangzhou, China). Its original name was ‘9311’. They are abbreviated B-2X and Y-2X hereafter, respectively. Tetraploid rice Balilla (2n = 4*x* = 48) and Yangdao 6 (2n = 4*x* = 48) were obtained through chromosome doubling by our research group and are abbreviated B-4X and Y-4X, respectively (Table S1). Chromosome identification mainly followed the method of Zhang et al., with some modifications [52]. In 2019, B-2X, B-4X, Y-2X, and Y-4X were planted in the polyploid rice base of Hubei University, Huangpi District, Wuhan City, Hubei Province, China. The plot dimension was 90 cm × 106.8 cm, with three replications in a randomized block design. The plant spacing was 10 cm × 26.7 cm, with one seedling per hill. The sowing, transplanting, and harvesting dates were May 20th, June 18th, and October 4th, respectively. All the materials were grown under natural conditions. Fertilizer and water were managed in accordance with popular management methods in Wuhan. After the ripening stage, three groups of 1000 plump seeds from each plot were sampled to measure the 1000 grain weights, and 30 plump seeds from each plot were sampled to measure the widths and lengths of brown rice. Seeds were harvested when fully ripe and placed in a freezer at −80 °C.

### 2.2. Reagents and Instruments

Methanol, acetonitrile, and acetic acid (HPLC/SPECTRO grade) were purchased from Merck, Germany. Deionized water was obtained using the Millipore purification system (Bedford, MA, USA), and lidocaine was purchased from BioBioPha Company (Kunming, China). An Ultra Performance Liquid Chromatograph (Shim-pack UFLC CBM20A) was purchased from Shimadzu (Kyoto, Japan). A tandem mass spectrometer (4500 Q TRAP) was purchased from Applied Biosystems (Framingham, MA, USA). The HSS T3 C18 column (100 mm × 2.1 mm × 1.8 µm) was purchased from Waters Corporation (Milford, MA, USA). The Himac CT6E High-Speed Centrifuge was purchased from Hitachi (Tokyo, Japan).

### 2.3. Sample Preparation and Extraction

Before the experiment, samples were numbered after rice hulling, crushed with a grinding machine at room temperature, placed in glass sample vials, and stored in a refrigerator at −80 °C. Three samples from each rice variety were tested. The cryopreserved brown rice samples were crushed using a mixer mill (MM 400, Retsch) with a zirconia bead for 1.5 min at 30 Hz. Then, 100 mg of each powdered sample was dissolved in 1.0 mL of extracting solution (70% methanol). The resulting mixtures were stored overnight at 4 °C and vortexed three times to ensure complete extraction. Following extraction, the pellets were centrifuged at 10,000× *g* for 10 min. The extracts were filtered through a microporous membrane (0.22-µm pore size) and stored in a sample vial. Quality control (QC) samples were prepared by mixing sample extracts. During analysis, a QC sample was included in the measurement queue for every three test samples to monitor measurement repeatability.

### 2.4. HPLC Conditions and ESI-Q TRAP-MS/MS

Chromatographic conditions for metabolites were optimized in accordance with Chen et al. [44] and Wang et al. [45]. HPLC conditions: The sample extracts were analyzed using an LC-ESI-MS/MS system mainly consisting of UFLC (Shim-pack UFLC Shimadzu CBM20A system, http://www.shimadzu.com.cn, accessed on 25 November 2019) and MS (Applied Biosystems 6500 Q TRAP, http://www.appliedbiosystems.com.cn, accessed on 25 November 2019). Analytical conditions were as follows. The HSS T3 C18 (100 mm × 2.1 mm × 1.8 µm) chromatographic column was used. Samples were rapidly eluted using 0.1% formic acid in water (solvent A) and 0.1% formic acid in acetonitrile (solvent B). Separation was achieved with the following gradients: starting with 5% solvent B, increased to 95% B in 11 min, kept at 95% B for 1 min, dropped quickly to 5% within 0.1 min, and kept at 5% B for 3 min. The constant flow rate was 0.4 mL/min, the column temperature was 40 °C, and the injection volume was 5 µL.

ESI-Q TRAP-MS/MS: Linear ion trap (LIT) and triple quadrupole (QqQ) scans were acquired on a QqQ-LIT mass spectrometer, API 6500 Q TRAP LC/MS/MS system, equipped with an ESI Turbo Ion-Spray interface, operating in positive ion mode and controlled by Analyst 1.6 software (AB Sciex). The ESI source operation parameters were as follows: an ion source, turbo spray; source temperature, 550 °C; ion-spray voltage, 5500 V; ion source gas I, gas II, and curtain gas set at 55, 60, and 25 psi, respectively; and collision gas, high. Instrument tuning and mass calibration were performed with 10 and 100 μmol/L polypropylene glycol solutions in QqQ and LIT modes, respectively. The QqQ scans were carried out as multiple reaction monitoring (MRM) experiments with collision gas (nitrogen) set to 5 psi. Declustering potential (DP) and collision energy (CE) for individual MRM transitions were used for further DP and CE optimization. A specific set of MRM transitions were monitored for each period according to the metabolites eluted within this period.

### 2.5. Qualitative and Quantitative Analysis of Metabolites

Qualitative analysis: Based on the Metware database (MVDB) and the metabolite information public database, qualitative analysis of primary and secondary mass spectrometry data were obtained by referencing existing MS databases such as MassBank (http://www.massbank.jp, accessed on 1 December 2019), KNAPSAcK (http://kanaya.naist.jp/KNApSAcK, accessed on 1 December 2019), Human Metabolome Database (http://www.hmdb.ca, accessed on 1 December 2019), MoTo DB (http://www.ab.wur.nl/moto, accessed on 1 December 2019), and METLIN (http://metlin.scripps.edu/index.php, accessed on 1 December 2019). The structural analysis of metabolites was determined.

Quantitative analysis: Metabolites were quantified via the MRM mode using QqQ MS. In the MRM mode with a detection window of 80 s and a target scan time of 1.5 s, the quadrupole filters the precursor ions (parent ions) of the target substance and excludes the ions corresponding to other molecular weights to prevent interference. After obtaining metabolite data from the different samples, the peak area of the mass spectra of all substances was integrated, and the mass spectra of the same metabolites in different samples were corrected.

### 2.6. Metabolite Data Analysis

Principal component analysis (PCA), system clustering and data standardization, pattern recognition, and metabolic network analysis of the diploid and tetraploid *japonica* rice metabolites were performed on the MetaboAnalyst 4.0 platform. Partial least squares-discriminant analysis (PLS-DA) was used to maximize the metabolome differences between the diploid and tetraploid rice samples. The relative importance of each metabolite to the PLS-DA model was checked using the parameter called variable importance in projection (VIP). Metabolites with VIP ≥ 1 and *p* ≤ 0.05 were considered as differential metabolites for group discrimination. A heatmap based on the hierarchical cluster analysis (HCA) method was produced using R software (www.r-project.org, accessed on 22 February 2020). Analysis of metabolic pathways was achieved using the Kyoto Encyclopedia of Genes and Genomes (KEGG) metabolic pathway database (http://www.genome.jp/kegg, accessed on 22 February 2020), metabolite set enrichment analysis or pathway analysis, and pathway topology analysis. The content of free fatty acids in brown rice was determined by spectrophotometry.

## 3. Results

### 3.1. Phenotype Comparison and Chromosome Identification of Diploid and Tetraploid Rice

**Figure 1:**
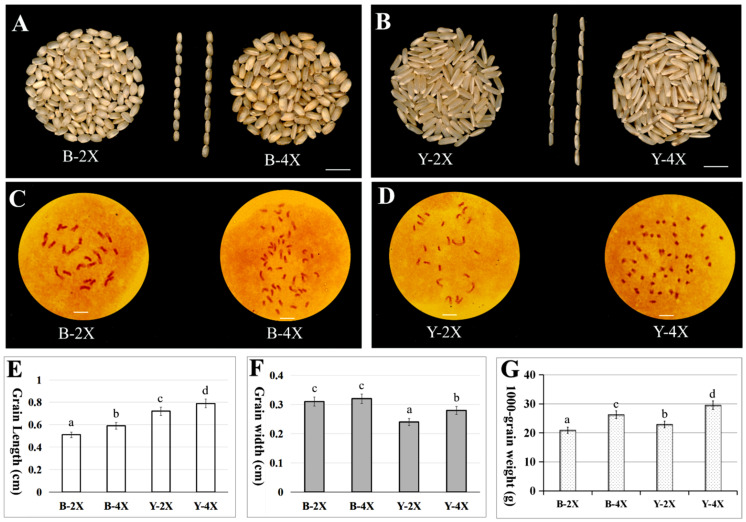
Brown rice comparison and chromosome identification between diploid and tetraploid rice. (**A**) Diploid brown rice of Balilla (B-2X) vs. tetraploid brown rice of Balilla (B-4X). In the middle, 10 B-2X (left) are compared with 10 B-4X (right) with respect to length. (**B**) Diploid brown rice of Yangdao 6 (Y-2X) vs. tetraploid brown rice of Yangdao 6 (Y-4X). In the middle, 10 Y-2X (left) are compared with 10 Y-4X (right) with respect to length. (**C**,**D**) Root tip chromosomes of rice. (**C**) B-2X: diploid Balilla (2n = 2*x* = 24); B-4X: tetraploid Balilla (2n  =  4*x*  =  48). (**D**) Y-2X: diploid Yangdao 6 (2n  =  2*x*  =  24); Y-4X: tetraploid Yangdao 6 (2n  = 4*x* = 48). (**E**–**G**) Comparison between diploid and tetraploid brown rice: (**E**) grain length, (**F**) grain width and (**G**) 1000-grain weight. Duncan’s multiple range test method was used for analysis. Different letters in the same column indicate significant differences (*p* < 0.05). *Bars* = 1 cm in (**A**,**B**), 5 μm in (**C**,**D**).

The phenotypes of diploid and tetraploid brown rice differed significantly. Tetraploid brown rice was larger (Figure 1A,B). Compared with diploid rice, the grain length and width of tetraploid *indica* rice Y-4X were both significantly increased, but the tetraploid *japonica* rice B-4X only showed a significant increase in grain length (Figure 1E,F). The 1000-grain weight of tetraploid brown rice was 25.56–28.94% higher than that of diploid brown rice (Figure 1G). Chromosome numbers in root tips of diploid and tetraploid plants were counted. Chromosomes of Balilla-2*x* and Yangdao 6-2*x* were 2n = 2*x* = 24, whereas those of Balilla-4*x* and Yangdao 6-4*x* were 2n = 4*x* = 48 (Figure 1C,D).

### 3.2. Widely Targeted Metabolic Profiling of Diploid and Tetraploid Brown Rice Based on LC-MS/MS

Two groups of diploid–tetraploid brown rice (Figure 1A,B), with three biological replicates, making a total of 12 samples, were used to portray the metabolic profiles employing the widely targeted metabolomics approach. A typical total ion current plot of one QC sample is shown in (Figure S1), which is the spectrum obtained by continuously summing the intensity of all ions in the mass spectrum at different time points. The multi-substance extracted ion chromatogram is usually used to determine the ion flux spectrum of each extracted substance in MRM mode (Dataset S1, Table S2). The multi-peak detection plot of metabolites in MRM mode is shown in Figure S2. Based on the MVDB and KEGG databases, and MRM, the qualitative and quantitative mass spectrometry analyses were performed on the metabolites in the samples. In total, 401 metabolites were identified, comprising 70 lipids, 68 amino acids and derivatives, 50 phenolic acids, 2 anthocyanins, 30 flavonoids, 9 flavonols, 22 flavonoid carbonosides, 2 isoflavones, 7 phenolamines, 22 alkaloids, 6 plumeranes, 33 nucleotides, and derivatives, 29 organic acids, 27 saccharides and alcohols, 7 vitamins, and 17 others (Table S3).

### 3.3. PCA for Diploid–Tetraploid Groups

**Figure 2:**
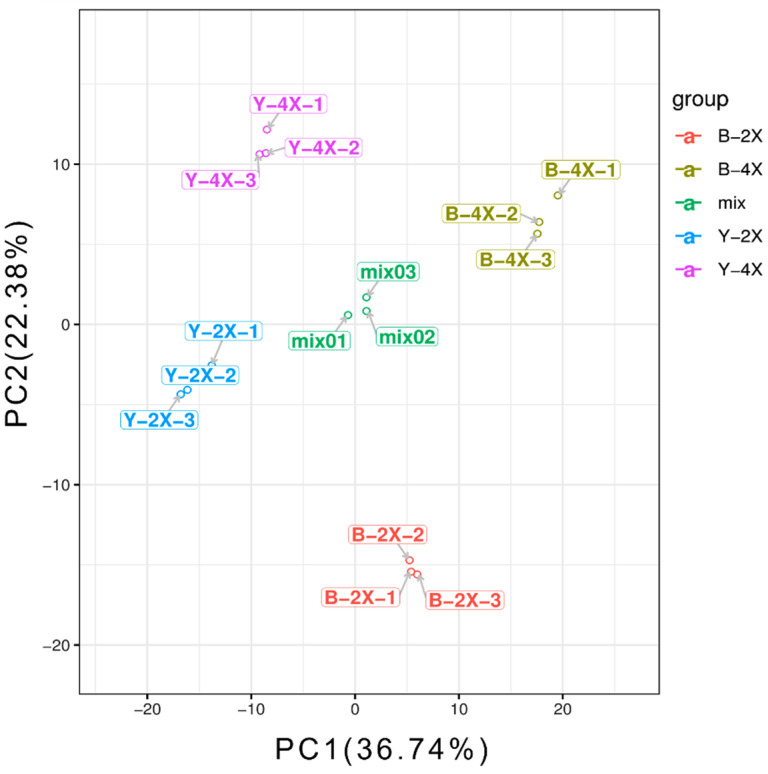
PCA scores plot map and hierarchical cluster analysis of diploid vs. tetraploid groups. (A) PCA scores plot for the diploid–tetraploid groups. B-2X: diploid Balilla, B-4X: tetraploid Balilla, Y-2X: diploid Yangdao 6, Y-4X: tetraploid Yangdao 6.

The PCA score scatter plots for all samples are shown in Figure 2, where the abscissa and the ordinate represent the PC1 and PC2 scores, respectively. The distinction between the diploid–tetraploid Balilla (*japonica*) group, the diploid–tetraploid Yangdao 6 (*indica*) group, and the mixed group was significant based on the top-ranking PCs; all the samples were within 95% confidence intervals (Hotelling’s T-squared ellipse). The values of one rice variety were clustered in the PCA score plot of metabolites, but the values of the different groups were separated. The PCA results suggested significant differences in metabolic phenotypes between each group.

### 3.4. Orthogonal Projections to Latent Structures–Discriminant Analysis (OPLS-DA) for Diploid vs. Tetraploid Groups

**Figure 3:**
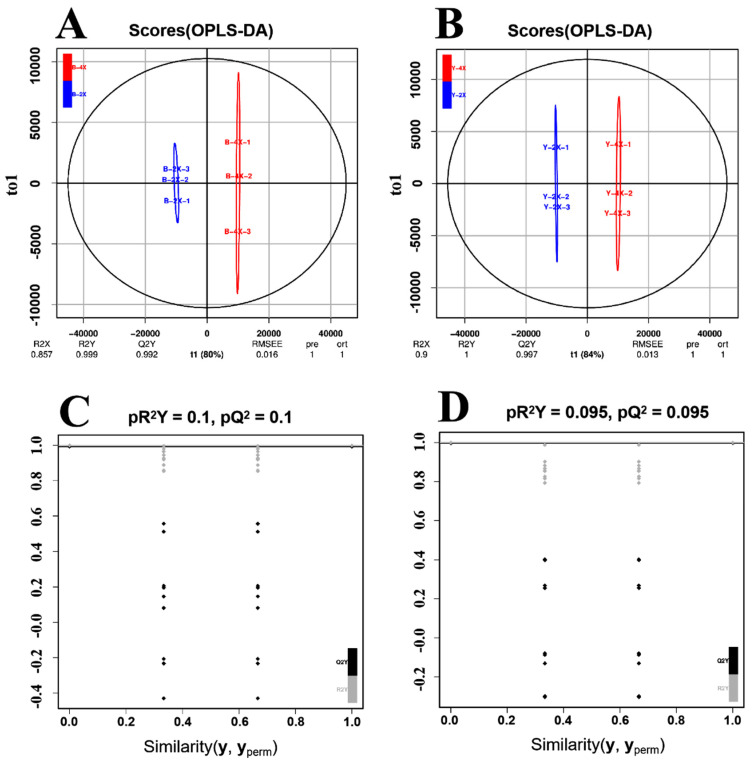
OPLS-DA scores and permutation verification. Scores of the OPLS-DA model with (**A**) Balilla-2*x* vs. Balilla-4*x* and (**B**) Yangdao 6-2*x* vs. Yangdao 6-4*x*. OPLS-DA permutation analysis model verification chart of (**C**) Balilla-2*x* vs. Balilla-4*x* and (**D**) Yangdao 6-2*x* vs. Yangdao 6-4*x*. R^2^Y and Q^2^ represent the interpretation rate of the model to the Y matrix and the prediction ability of the model, respectively. A value closer to 1 means that the model is more stable and reliable, and when Q^2^ > 0.9, the model is excellent. The horizontal line corresponds to the R^2^ and Q^2^ of the original model, and the black and gray points represent the R^2^, and Q^2^ of the model after Y replacement, respectively.

Compared with PCA, OPLS-DA can maximize the distinction between groups and is more conducive to finding differential metabolites. The scatter score plots inferred from the inter-group comparison of the diploid–tetraploid Balilla group and the diploid–tetraploid Yangdao 6 group in OPLS-DA are shown in Figure 3A,B, respectively. The R^2^Y and Q^2^Y scores were all greater than 0.99 in the B-2*x* vs. B-4*x* (Figure 3A) and Y-2*x* vs. Y-4*x* (Figure 3B), demonstrating that the ploidy difference led to the differential metabolism. The OPLS-DA model was established using many (*n* = 200) alignment experiments. The horizontal line corresponds to the R^2^ and Q^2^ of the original model, and the black and gray points represent the R^2^ and Q^2^ of the model after Y replacement, respectively (Figure 3C,D). The stable and reproducible model provided a satisfactory explanation of the difference between the two groups of samples. The OPLS-DA results showed that the differential metabolites could be screened according to VIP value in the subsequent analysis.

### 3.5. HCA and Volcano Plot of Differential Metabolites for Diploid vs. Tetraploid Groups

The HCA can classify metabolites with the same characteristics and identify the differences between groups. Therefore, it can be used to evaluate the characteristic difference of metabolite accumulation caused by ploidy differences. The HCA plot of the differential metabolites identified in comparing the diploid with the tetraploid groups is shown in Figure S3. The HCA results showed a clear grouping pattern of different species.

Differential metabolites were also analyzed using volcano plots (Figure S4). The points in the volcano plot represent the metabolites, the abscissa indicates the fold change (FC = 4X/2X) (log_2_) of each substance in the group, and the ordinate indicates the *p*-values (log_10_) of Student’s *t*-test. Metabolites with FC of ≥ 2 or FC ≤ 0.5, and *p* < 0.05 were selected. In the *japonica* group, there were 182 higher-abundance and 56 lower-abundance metabolites in the tetraploid compared with diploid. In the *indica* group, there were 86 higher-abundance and 120 lower-abundance metabolites in the tetraploid compared with diploid.

**Figure 4:**
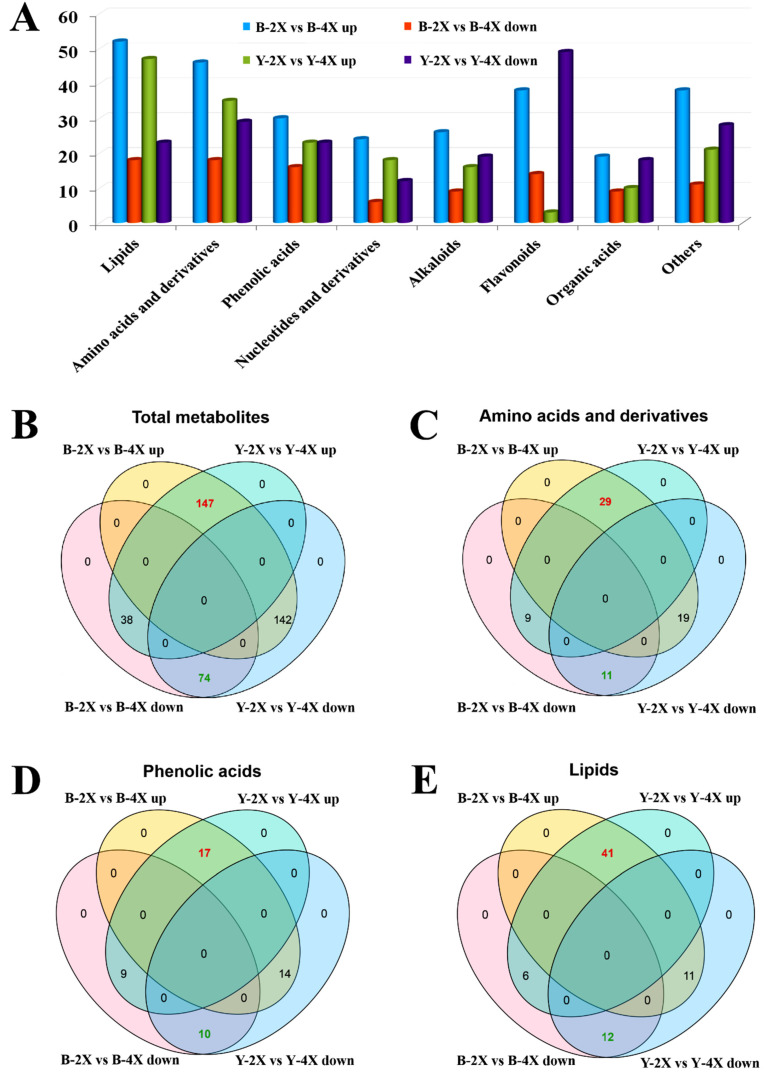
Number of different types of differential metabolites for diploid vs. tetraploid groups. (**A**) Number of different types of differential metabolites for diploid vs. tetraploid. Blue color indicates the number of higher-abundance metabolites of Balilla-2*x* vs. Balilla-4*x*, and orange color indicates the number of lower-abundance metabolites. Green color indicates the number of higher-abundance metabolites of Yangdao 6-2*x* vs. Yangdao 6-4*x*, and purple indicates the number of lower-abundance metabolites. (**B**–**E**) Comparison of expression trends of differential metabolites for diploid vs. tetraploid between *japonica* and *indica* groups. (**B**) Total metabolites, (**C**) amino acids and derivatives, (**D**) phenolic acids, (**E**) lipids.

The FC can describe the changes from initial to final values. In the current study, log_2_ FC was used to analyze the relative expression changes of metabolites between diploid and tetraploid brown rice. If log_2_ FC > 0, then the expression abundance of the metabolite in tetraploid brown rice was higher; if log_2_ FC < 0, then the expression abundance was lower. Of the 401 metabolites identified between the *japonica* and *indica* groups, 221 showed the same trends (147 in higher abundance vs. 74 in lower abundance) (Figure 4A,B). Moreover, the numbers of higher expression abundance metabolites of lipids, amino acids and derivatives, and phenolic acids in tetraploid rice were significantly increased in both the *japonica* and *indica* groups (Figure 4A,C–E). In particular, 53 of the 70 identified lipid differential metabolites showed the same trend between *japonica* and *indica* rice groups. Among them, 77.36% (41 out of 53) had high expression abundances in tetraploid rice (Figure 4E).

### 3.6. Clustering, Pathway, and Enrichment Analyses of Lipid Metabolites for Diploid vs. Tetraploid Groups

**Figure 5:**
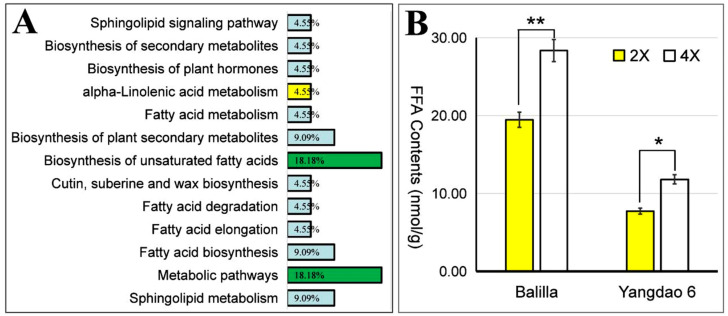
Pathway analysis of lipid metabolites for diploid vs. tetraploid groups and determination of free fatty acids in brown rice. (**A**) Functional annotation and KEGG classification of lipid metabolites. The ordinate is the name of the KEGG metabolic pathway, the abscissa is the number of metabolites annotated to the pathway and its proportion in the total number of lipid metabolites annotated. The proportions of “Biosynthesis of unsaturated fatty acids” and “Metabolic pathways” were both 18% (green background). In addition, the proportion of “alpha-Linolenic acid metabolism” was 4.55% (yellow background). (**B**) Determination of free fatty acids in diploid (2X, yellow) and tetraploid (4X, white) brown rice. * Significantly different at 0.05 probability level. ** Significantly different at 0.01 probability level.

The above analysis showed that most (levels of 77.36%) lipid metabolites were present in higher abundances. The heatmap of lipid metabolite changes for the diploid vs. tetraploid groups is shown in Figure S5. The results showed a clear grouping pattern of different species. The most differential lipid metabolites identified in the study showed similar positively or negatively regulated trends between *japonica* and *indica* groups, consistent with the previous analysis. The results of these lipid metabolite annotations were classified according to the pathway types in the KEGG database, revealing that 13 metabolic pathways were involved (Figure 5A). Among these pathways, “Metabolic pathways” refers to an overall metabolic pathway in plants, covering most known metabolites. Therefore, this pathway was usually involved. In addition, another pathway “Biosynthesis of unsaturated fatty acids” was also involved in this analysis. This indicates that the increase in ploidy mainly changed the content of the unsaturated fatty acids of brown rice.

Studies have shown that the main free fatty acids in rice are unsaturated fatty acids [10]. To verify this previous conclusion, we determined the content of free fatty acids in brown rice (Figure 5B). The content of free fatty acids in Balilla-4*x* was 28.36 nmol/g, which was 45.66% higher than in Balilla-2*x* (19.47 nmol/g), and the content in Yangdao 6-4*x* was 11.82 nmol/g, which was 52.91% higher than that in Yangdao 6-2*x* (7.73 nmol/g). Thus, the content of free fatty acids mainly composed of unsaturated fatty acids increased significantly in tetraploid rice, which is consistent with previous analysis.

### 3.7. Statistical Analysis of Significant Differential Lipid Metabolites for Diploid vs. Tetraploid Groups

In the current study, the VIP and *p*-value were used to analyze the significant differential lipid metabolites. If VIP > 1 and *p* < 0.01, a significant difference in the metabolite exists between the diploid and tetraploid groups. There were 11 metabolites with significant differences (Table 1): two free fatty acids (γ-linolenic and punicic acids), five lysophosphatidylcholines (LysoPC 15:0, LysoPC 16:1, LysoPC 18:1, LysoPC 18:3, and LysoPC 18:3 2n isomer), two glycerol esters (MAG 18:2 and MAG 18:3 isomer1), one sphingolipid (4-hydroxysphinganine), and one phosphatidylcholine (choline alfoscerate). Most of these metabolites had significantly higher abundances in tetraploid rice (Table 1). Among them, all the lysophosphatidylcholines increased significantly and may be important contributors to the increase of PL content in tetraploid rice. Among the annotated free fatty acid metabolites, the level of γ-linolenic increased significantly and indicated that up-regulation of γ-linolenic acid may play an important role in the increase of unsaturated fatty acids in tetraploid rice.

**Figure 6:**
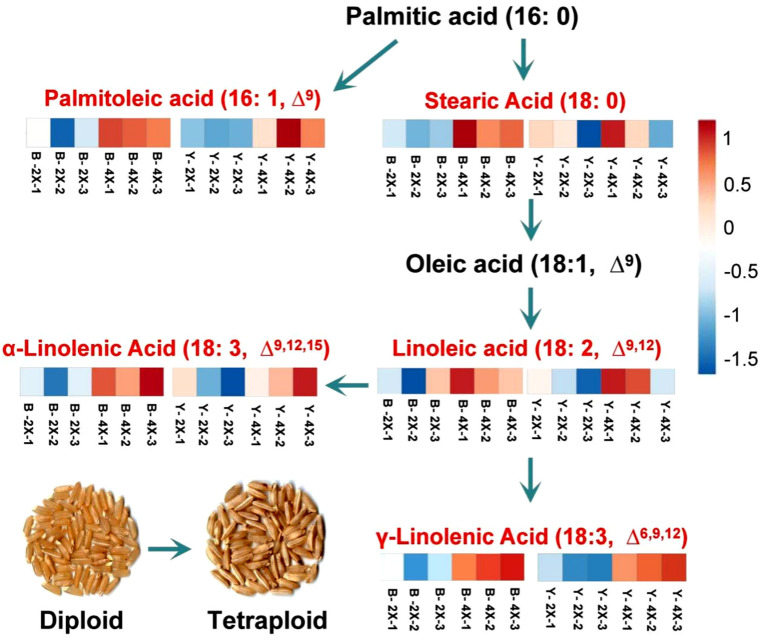
Schematic presentation of the pathway for certain unsaturated fatty acids metabolites as affected by polyploidization in rice. Red represents increases. The checkerboard is the heatmap of metabolites for the diploid vs. tetraploid groups. Ratios of fold changes are given by shades of red or blue color according to the scale bar.

The γ-linolenic acid is formed by the elongation and desaturation of a palmitic acid carbon chain. The pathways for the synthesis of certain unsaturated fatty acids, as affected by rice polyploidization, are shown in Figure 6. The heatmap and log_2_ FC value of lipid metabolite changes between diploid and tetraploid brown rice indicated that the expression abundances of palmitoleic (16:1, ∆^9^), stearic (18:0), linoleic (18:2, ∆^9, 12^), α-linolenic (18:3, ∆^9, 12, 15^), and γ-linolenic (18:3, ∆^6, 9, 12^) acids increased. These unsaturated fatty acids are the main components of free fatty acids in rice. This indicated that the expression abundances of palmitoleic acid, stearic acid, linoleic acid, α-linolenic acid, and especially γ-linolenic acid in tetraploid rice were up-regulated after polyploidization. Finally, the content of free fatty acids in tetraploid rice increased significantly.

## 4. Discussion

### 4.1. Differential Expression of Metabolites in Diploid and Tetraploid Rice

Metabolites in seeds are important for nutritional quality. Thus, metabonomics has been often used to analyze seed nutrients. In the past, using chemical methods, researchers compared nutrients between diploid and autotetraploid rice, such as amino acids, proteins, and starches [25,53]. However, there have been no reports on comparative analyses of secondary metabolomics in diploid and tetraploid rice. In this study, 401 metabolites of two groups of diploid–tetraploid brown rice were identified based on LC-MS/MS. The analysis revealed very different expression abundances of metabolites between diploid and tetraploid groups. In particular, the number of lipids, amino acids and derivatives, and phenolic acids with high abundance expression levels in tetraploid rice increased significantly. The result once again confirmed that the amino acid content would increase significantly after rice polyploidization. Additionally, for the first time, we identified that the expression abundances of lipids (e.g., unsaturated fatty acids and PLs) and phenolic acids also increased significantly. In particular, 77.36% of lipid metabolites in tetraploid rice showed higher expression abundances than in diploid rice. These identified lipid metabolites were classified in pathways of the KEGG database. One of the highly involved pathways was “biosynthesis of unsaturated fatty acids”. Among the lipid metabolites with significant differential expression levels (VIP > 1, *p* < 0.01) between diploid and tetraploid rice, the abundances of γ-linolenic acid, LysoPC 15:0, LysoPC 16:1, LysoPC 18:1, LysoPC 18:3, and LysoPC 18:3 2n isomers in tetraploid were significantly higher. Further analyses indicated that the expression level of γ-linolenic acid increased significantly, and the expression abundances of most other unsaturated fatty acids in the γ-linolenic acid synthesis pathway also increased. Therefore, we speculated that the high abundance expressions of most unsaturated fatty acid metabolites (especially γ-linolenic acid) and PL metabolites (especially the five LysoPCs) were important contributors to the increase in lipids after rice polyploidization.

### 4.2. Application Potential of Polyploid Rice in Functional Rice Breeding

Polyploidization usually leads to changes in plant phenotype and nutrition [37]. Rice polyploidization significantly changes the rice phenotype. Tetraploid rice seeds often become longer, wider, and heavier. At the same time, the chalkiness degree, chalkiness rate, gel consistency, alkali spreading value, and gelatinization temperature also changed [53]. Among nutrient substances, the amylose content often decreases in tetraploid rice, but the protein and amino acid contents increase [25,53,54,55]. Recent studies have shown that rice polyploidization can increase glutelin content by influencing glutelin biosynthesis transport, and deposition, to affect the contents of related essential amino acids [39]. Our study showed that the expression abundances of lipids (e.g., unsaturated fatty acids and PLs) and phenolic acids in tetraploid rice also increased significantly. The above nutrient changes in rice caused by polyploidization presented both opportunities and challenges to rice breeding research. On the one hand, many nutrients in rice are very beneficial to human health. For example, the glutelins in rice seeds are high-quality plant proteins containing several essential amino acids (such as lysine and arginine) that can be easily digested and absorbed [56,57]. Most of the free fatty acids in rice are high-quality unsaturated fatty acids, which benefit human health. Dietary PLs in rice help prevent diabetes, coronary heart disease, inflammation, cancer, and other chronic diseases. Additionally, the linolenic and linoleic acids have obvious effects in preventing arteriosclerosis and hypercholesterolemia [14,17,58]. The phenolic compounds of rice grains can eliminate the cell damage caused by free radicals. They have important anti-aging, cardiovascular disease prevention, and anti-cancer functions [59,60]. However, on the other hand, protein is often negatively correlated with cooking- and eating-quality rice. Rice with a high protein content has low viscosity, high hardness, and poor taste [61,62]. At the same time, rice with a high glutenin content is not beneficial to some people, such as kidney or diabetes patients. However, rice with a low glutelin content is good for their health [63]. In addition, lipids are easily hydrolyzed and oxidized during rice storage, which promotes rice aging and deterioration and leads to the decline in the edible quality. Thus, rice with a high lipid content is not resistant to storage [64]. How do breeders coordinate these two aspects? One way is to breed functional rice. Functional rice has some special nutritional components, such as functional proteins, oils, vitamins, flavonoids, essential trace elements, and functional and essential amino acids [65]. It is beneficial to human health, and its production is becoming an important direction for rice breeding [66]. A primary characteristic of functional rice being rich in certain target functional substances [67]. Thus, polyploid rice has great potential in functional rice breeding. Studies have shown that polyploidization affects the nutrient content in rice, such as significantly increasing the protein, amino acid, lipid, and phenolic acid contents. It will be valuable to cultivate functional rice varieties with high lipid, phenolic acid, or amino acid contents through polyploidization.

Autotetraploid rice was first reported in 1933 [68] and has a history of nearly 90 years. However, the low-fertility autotetraploid rice has always been difficult to use in agriculture. In recent years, the polyploid meiotic stability tetraploid rice lines and neo-tetraploid rice lines have broken through this limitation [27,30,35,69]. These high-seed-fertility tetraploid rice lines make it possible to breed nutrient-rich varieties.

## 5. Conclusions

The present study used widely targeted metabolites based on the LC-MS/MS detection platform to analyze the metabolic differences in diploid and tetraploid rice. This is the first analysis of the effects of rice polyploidization on nutritional quality from the perspective of metabonomics. Among the 401 metabolites identified, the expression abundances of lipids, amino acids and derivatives, and phenolic acids increased in tetraploid rice. In particular, the lipid contents, especially of unsaturated fatty acids and PLs, increased significantly after rice polyploidization, which has not been reported before. Overall, the present work provides more knowledge concerning the changes in rice nutritional quality after polyploidization, and more references for breeding eutrophic or functional rice, such as γ-linolenic acid-rich or LysoPCs-rich polyploid rice varieties.

## Figures and Tables

**Table 1:** Identification of significantly different lipid metabolites in diploid and tetraploid groups based on the criteria of VIP > 1 and *p* < 0.01.

Lipid Compounds	B-2*x* vs. B-4*x*			Y-2*x* vs. Y-4*x*		
	VIP	*p*	Trend	VIP	*p*	Trend
γ-Linolenic acid	2.13	1.85 × 10^−3^	up	1.11	8.38 × 10^−4^	up
Punicic acid	2.22	4.29 × 10^−3^	down	1.55	4.28 × 10^−4^	down
LysoPC (15:0)	1.28	3.73 × 10^−4^	up	1.25	3.94 × 10^−4^	up
LysoPC (16:1)	1.67	8.71 × 10^−4^	up	1.76	3.73 × 10^−4^	up
LysoPC (18:1)	2.50	9.17 × 10^−4^	up	2.31	1.15 × 10^−3^	up
LysoPC (18:3)	5.62	4.16 × 10^−5^	up	2.82	1.97 × 10^−3^	up
LysoPC (18:3) (2n isomer)	5.48	2.69 × 10^−4^	up	3.02	2.54 × 10^−3^	up
4-Hydroxysphinganine	1.07	6.91 × 10^−4^	down	1.82	1.74 × 10^−3^	up
Choline alfoscerate	1.34	6.37 × 10^−3^	up	1.39	7.18 × 10^−4^	up
MAG (18:2)	5.34	2.33 × 10^−4^	up	4.83	9.12 × 10^−5^	up
MAG (18:3) isomer1	2.26	1.89 × 10^−4^	up	1.08	1.13 × 10^−4^	up

Note: the “up” and “down” represent higher and lower abundances of lipid metabolites in tetraploid rice compared with diploid rice, respectively.

## Data Availability

Based on the Metware database (MVDB) and the metabolite information public database, qualitative analysis of primary and secondary mass spectrometry data were obtained by referencing existing MS databases such as MassBank (http://www.massbank.jp, accessed on 17 December 2019), KNAPSAcK (http://kanaya.naist.jp/KNApSAcK, accessed on 17 December 2019), Human Metabolome Database (http://www.hmdb.ca, accessed on 17 December 2019), MoTo DB (http://www.ab.wur.nl/moto, accessed on 17 December 2019), and METLIN (http://metlin.scripps.edu/index.php, accessed on 17 December 2019). The structural analysis of metabolites was determined.

## Supplementary Materials

The following are available online at https://www.mdpi.com/article/10.3390/plants11010132/s1, Table S1: Classification and phenotypes of the 4 rice cultivars used for metabolomics. Table S2: Retention time of 11 representative metabolites. Table S3: The sample data. Figure S1: Detection of the TIC overlap map by QC sample mass spectrometry. Figure S2: MRM metabolite detection multipeak map. Figure S3: Hierarchical cluster analysis of diploid vs. tetraploid groups. The red segments indicate a relatively high content of metabolites, while the green segments indicate a relatively low content of metabolites. Figure S4: Volcano plot of (A) Balilla-2x vs. Balilla-4x and (B) Yangdao 6-2x vs. Yangdao 6-4x. The colors of the scatter points indicate the final screening results: red indicates metabolites that were significantly higher in abundance, green indicates metabolites that were significantly lower in abundance, and blue indicates metabolites with non-significant difference. Figure S5: Heatmap of lipid metabolite changes in brown rice grains. Red segments indicate a relatively high content of metabolites, and green segments indicate a relatively low content of metabolites. Dataset S1: MS2 spectra of 11 representative metabolites.
