# Supplementary material for: Polyploidization Increases the Lipid Content and Improves the Nutritional Quality of Rice
Source: Plants (Basel). 2022 Jan 4;11(1):132. doi: 10.3390/plants11010132 (PMC8747249; doi:10.3390/plants11010132)
Supplement: Supplementary file 1 [file plants-11-00132-s001.zip › plants-1499009-supplementary/Supplementary files-Final/Additional file 1-Supplementary Tables/Supplementary Table S1 (Classification and phenotypes).pdf]

Supplementary Table S1. Classification and phenotypes of the 4 rice cultivars used for metabolomics

Cultivar	Ploidy	Grain Type	Bran Color	Country of Origin	Growing Location
Balilla	Diploid	Short	Brown	Italy	China
Tetraploid Balilla	Tetraploid	Short	Brown	China	China
Yangdao 6	Diploid	Long	Brown	China	China
Tetraploid Yangdao 6	Tetraploid	Long	Brown	China	China

Abbreviations in the text: ① B-2X: Balilla-2X; ② B-4X: Balilla-4X; ③ Y-2X: Yangdao 6-2X; ④ Y-4X: Yangdao 6-4X.
