# Supplementary figures and images for: Polyploidization Increases the Lipid Content and Improves the Nutritional Quality of Rice
Source: Plants (Basel). 2022 Jan 4;11(1):132. doi: 10.3390/plants11010132 (PMC8747249; doi:10.3390/plants11010132)

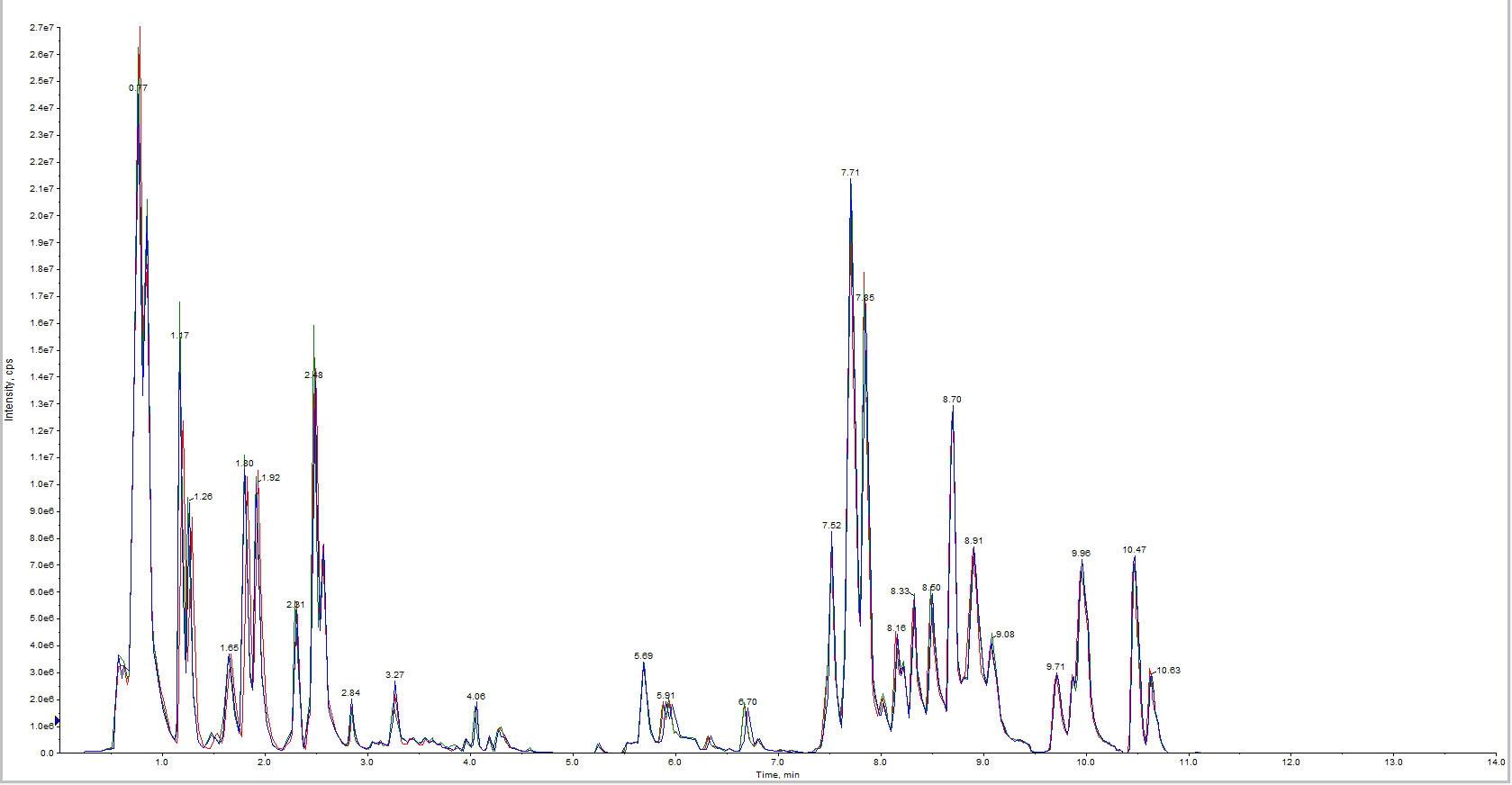

Supplement: Supplementary file 1 [file plants-11-00132-s001.zip › plants-1499009-supplementary/Supplementary files-Final/Additional file 2-Supplementary Figures/Supplementary Figure S1.png]

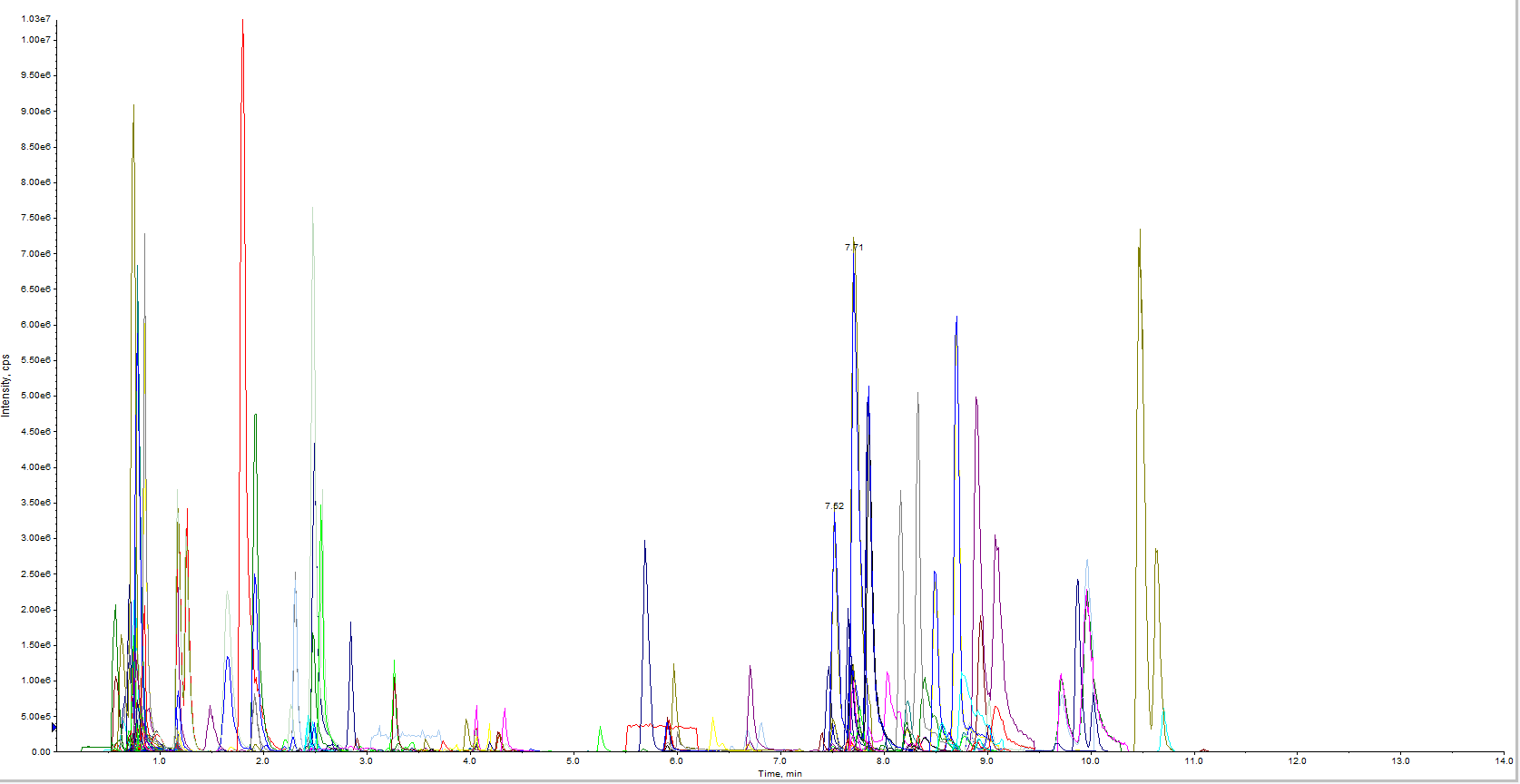

Supplement: Supplementary file 1 [file plants-11-00132-s001.zip › plants-1499009-supplementary/Supplementary files-Final/Additional file 2-Supplementary Figures/Supplementary Figure S2.png]

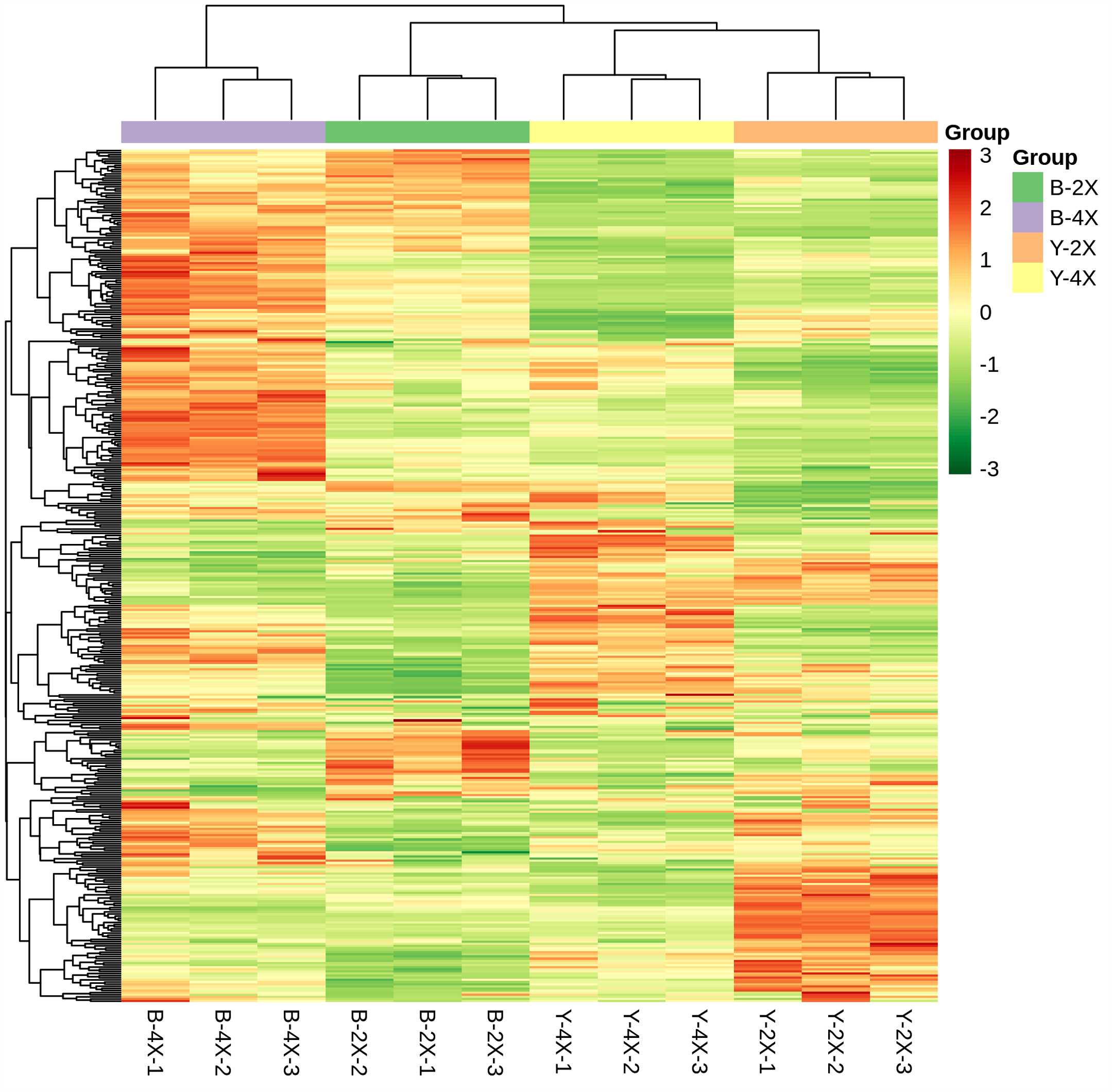

Supplement: Supplementary file 1 [file plants-11-00132-s001.zip › plants-1499009-supplementary/Supplementary files-Final/Additional file 2-Supplementary Figures/Supplementary Figure S3.png]

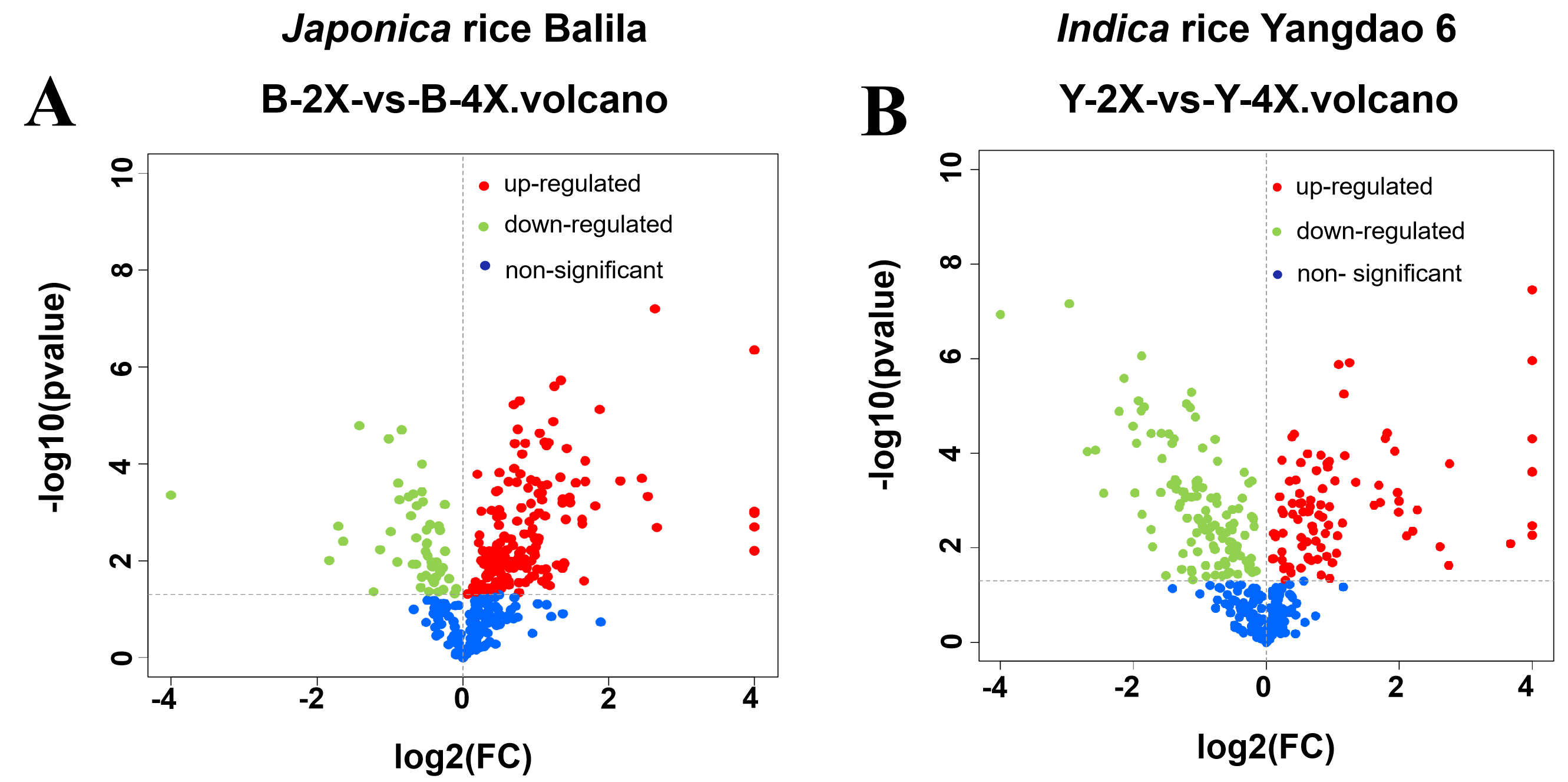

Supplement: Supplementary file 1 [file plants-11-00132-s001.zip › plants-1499009-supplementary/Supplementary files-Final/Additional file 2-Supplementary Figures/Supplementary Figure S4.png]

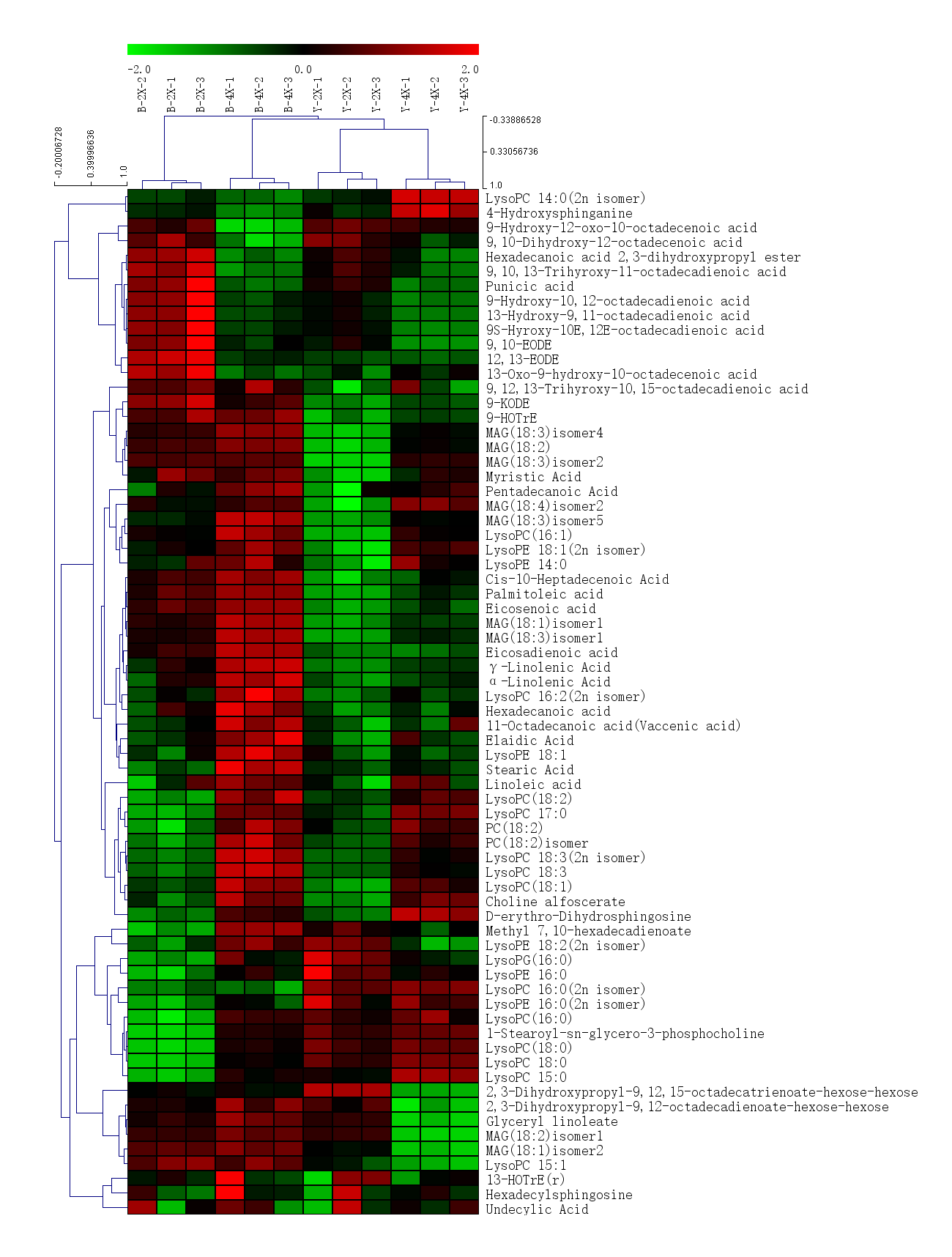

Supplement: Supplementary file 1 [file plants-11-00132-s001.zip › plants-1499009-supplementary/Supplementary files-Final/Additional file 2-Supplementary Figures/Supplementary Figure S5.bmp]

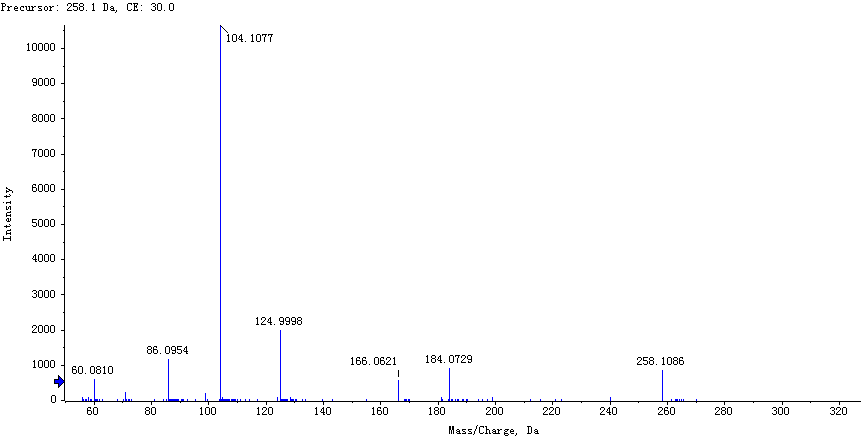

Supplement: Supplementary file 1 [file plants-11-00132-s001.zip › plants-1499009-supplementary/Supplementary files-Final/Additional file 3-Supplementary Dataset/mws0120 (Choline alfoscerate).png]

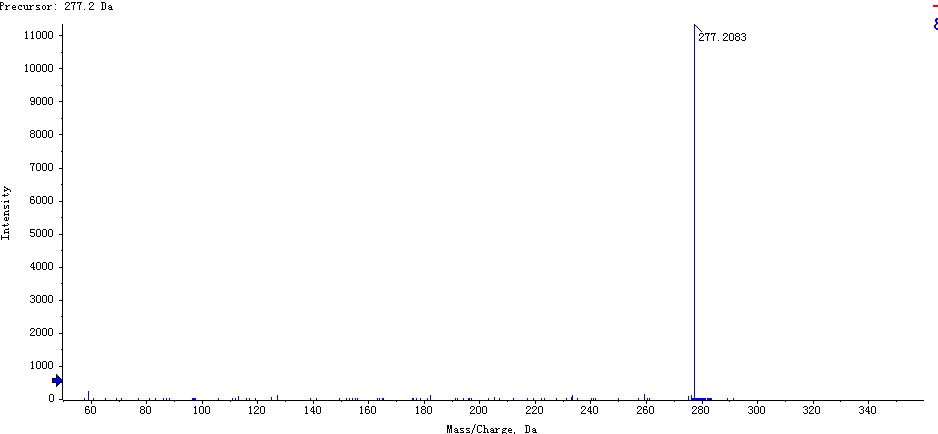

Supplement: Supplementary file 1 [file plants-11-00132-s001.zip › plants-1499009-supplementary/Supplementary files-Final/Additional file 3-Supplementary Dataset/mws0366 (a├-Linolenic Acid).png]

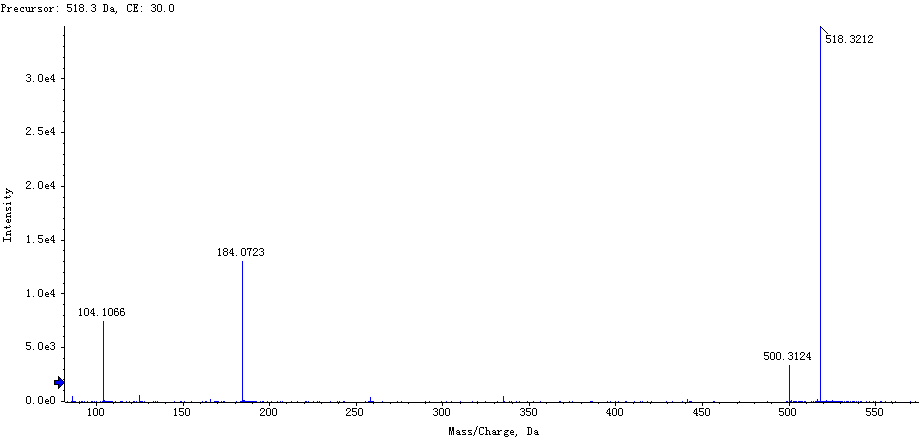

Supplement: Supplementary file 1 [file plants-11-00132-s001.zip › plants-1499009-supplementary/Supplementary files-Final/Additional file 3-Supplementary Dataset/pmb0854 (LysoPC 18-3).png]

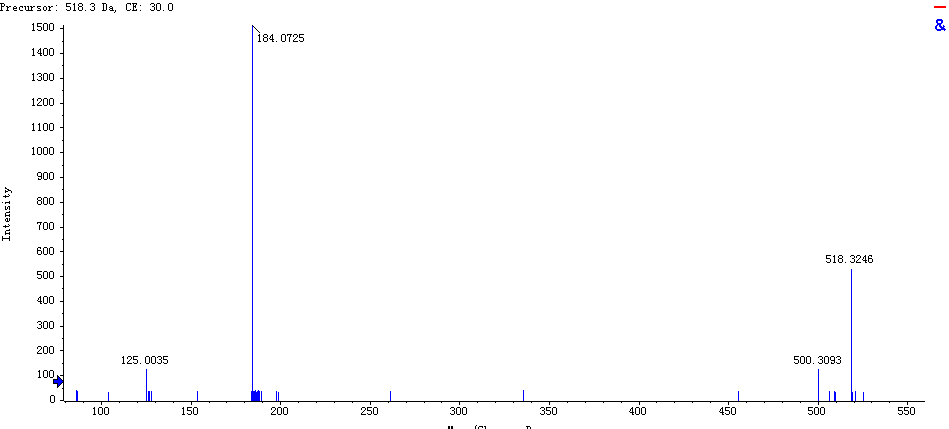

Supplement: Supplementary file 1 [file plants-11-00132-s001.zip › plants-1499009-supplementary/Supplementary files-Final/Additional file 3-Supplementary Dataset/pmb0865(LysoPC 18-3(2n isomer)).png]

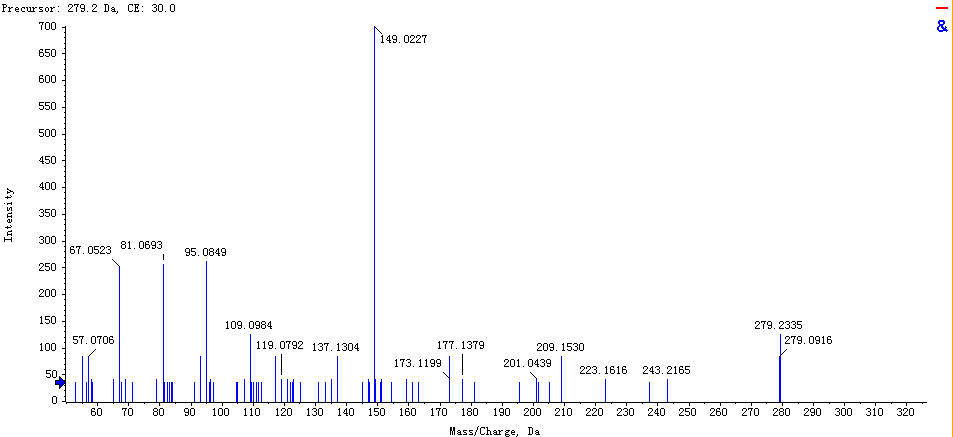

Supplement: Supplementary file 1 [file plants-11-00132-s001.zip › plants-1499009-supplementary/Supplementary files-Final/Additional file 3-Supplementary Dataset/pmb0889(Punicic acid).png]

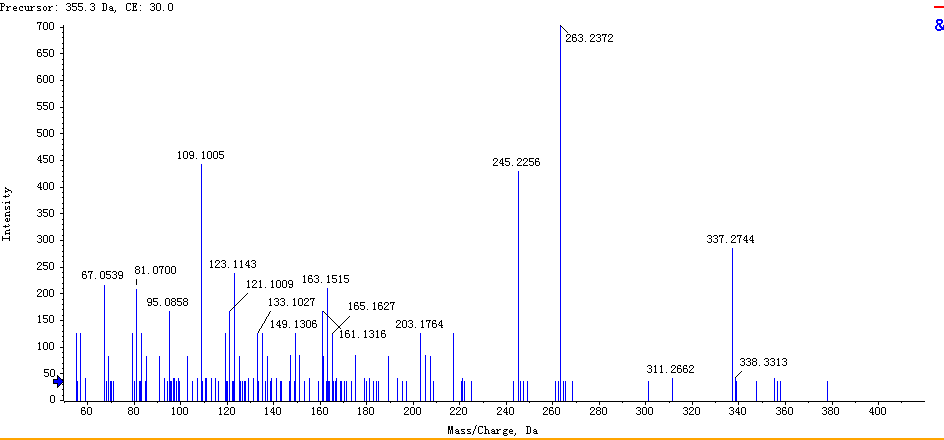

Supplement: Supplementary file 1 [file plants-11-00132-s001.zip › plants-1499009-supplementary/Supplementary files-Final/Additional file 3-Supplementary Dataset/pmb0890 (MAG(18-2)).png]

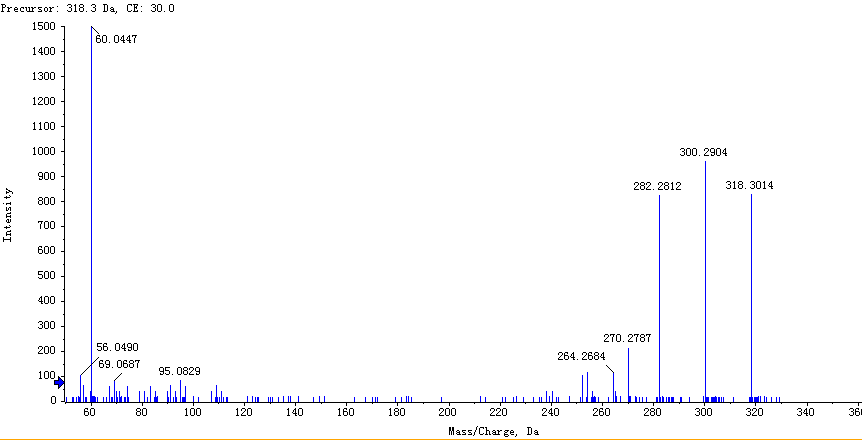

Supplement: Supplementary file 1 [file plants-11-00132-s001.zip › plants-1499009-supplementary/Supplementary files-Final/Additional file 3-Supplementary Dataset/pmb2221(4-Hydroxysphinganine).png]

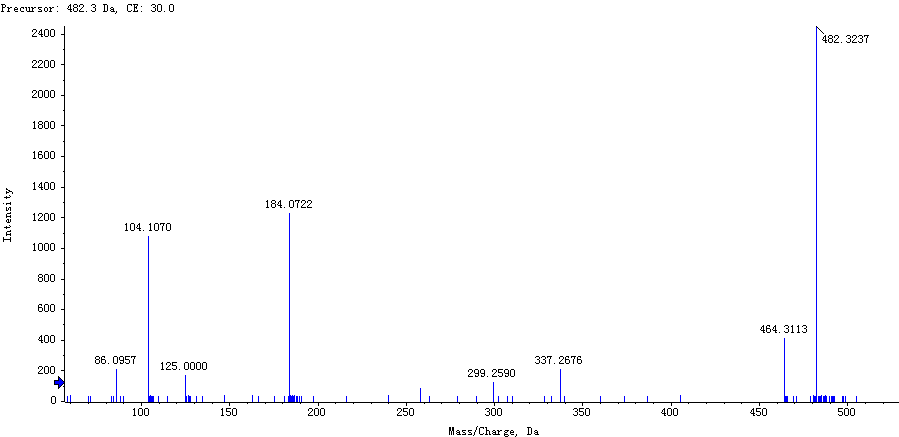

Supplement: Supplementary file 1 [file plants-11-00132-s001.zip › plants-1499009-supplementary/Supplementary files-Final/Additional file 3-Supplementary Dataset/pmb2319 (LysoPC 15-0).png]

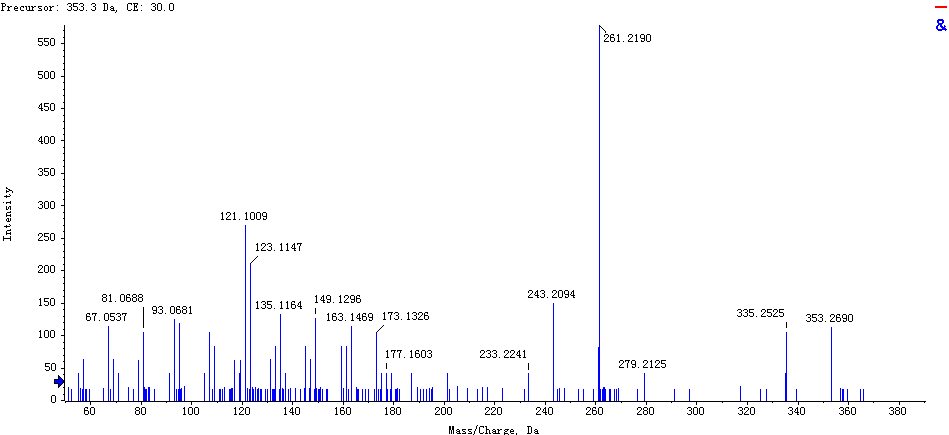

Supplement: Supplementary file 1 [file plants-11-00132-s001.zip › plants-1499009-supplementary/Supplementary files-Final/Additional file 3-Supplementary Dataset/pmb2444 (MAG(18-3)isomer1).png]

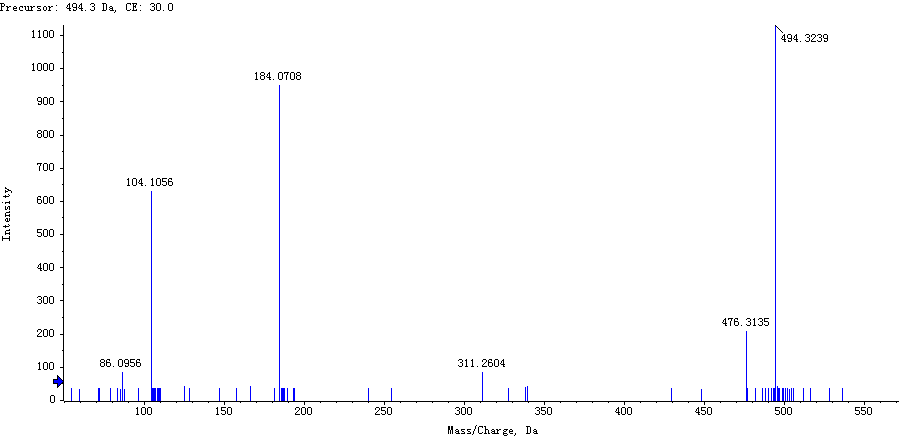

Supplement: Supplementary file 1 [file plants-11-00132-s001.zip › plants-1499009-supplementary/Supplementary files-Final/Additional file 3-Supplementary Dataset/pmp001270 (LysoPC(16-1)).png]

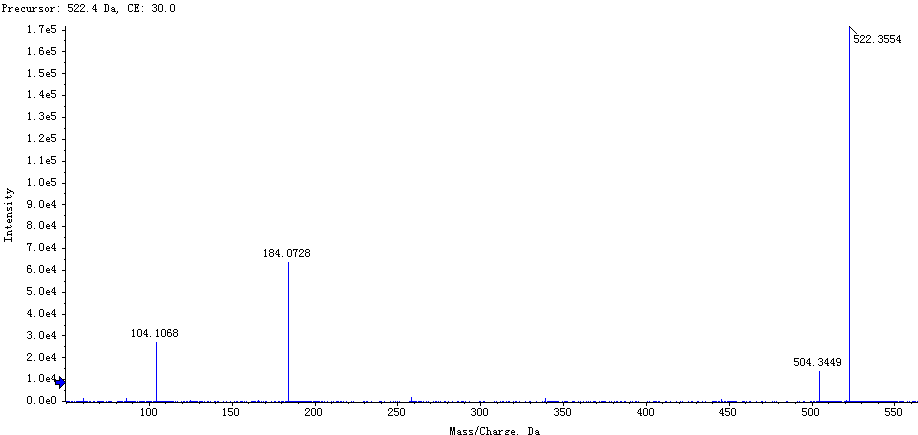

Supplement: Supplementary file 1 [file plants-11-00132-s001.zip › plants-1499009-supplementary/Supplementary files-Final/Additional file 3-Supplementary Dataset/pmp001281 (LysoPC(18-1)).png]
